# Current state of laser synthesis of metal and alloy nanoparticles as ligand-free reference materials for nano-toxicological assays

**DOI:** 10.3762/bjnano.5.165

**Published:** 2014-09-12

**Authors:** Christoph Rehbock, Jurij Jakobi, Lisa Gamrad, Selina van der Meer, Daniela Tiedemann, Ulrike Taylor, Wilfried Kues, Detlef Rath, Stephan Barcikowski

**Affiliations:** 1 Technical Chemistry I and Center for Nanointegration Duisburg-Essen (CENIDE), Universitaetsstr. 7, 45141 Essen, Germany; 2 Institute for Farm Animal Genetics, Friedrich-Loeffler-Institut, Höltystr. 10, 31535 Neustadt, Germany

**Keywords:** albumin, gold-silver, implant alloy, laser ablation, nickel-titanium, size control, wear debris

## Abstract

Due to the abundance of nanomaterials in medical devices and everyday products, toxicological effects related to nanoparticles released from these materials, e.g., by mechanical wear, are a growing matter of concern. Unfortunately, appropriate nanoparticles required for systematic toxicological evaluation of these materials are still lacking. Here, the ubiquitous presence of surface ligands, remaining from chemical synthesis are a major drawback as these organic residues may cause cross-contaminations in toxicological studies. Nanoparticles synthesized by pulsed laser ablation in liquid are a promising alternative as this synthesis route provides totally ligand-free nanoparticles. The first part of this article reviews recent methods that allow the size control of laser-fabricated nanoparticles, focusing on laser post irradiation, delayed bioconjugation and in situ size quenching by low salinity electrolytes. Subsequent or parallel applications of these methods enable precise tuning of the particle diameters in a regime from 4–400 nm without utilization of any artificial surface ligands. The second paragraph of this article highlights the recent progress concerning the synthesis of composition controlled alloy nanoparticles by laser ablation in liquids. Here, binary and ternary alloy nanoparticles with totally homogeneous elemental distribution could be fabricated and the composition of these particles closely resembled bulk implant material. Finally, the model AuAg was used to systematically evaluate composition related toxicological effects of alloy nanoparticles. Here Ag^+^ ion release is identified as the most probable mechanism of toxicity when recent toxicological studies with gametes, mammalian cells and bacteria are considered.

## Introduction

The widespread use of medical implants consisting of metals (e.g., gold coatings [1]) and alloys (e.g., NiTi, CoCr, stainless steel) [2–4] makes an adequate assessment of their toxicity a major issue, particularly as implants are designed to remain in contact with biological systems for years. Toxicological effects of implant materials have been examined for about two decades [5] and are most likely attributed to the release of micro- and nanoscopic wear debris [6–8] which have been reported to accumulate in lymph nodes, bone marrow, liver and spleen [9]. In that context toxicological effects, including impaired DNA replication and cell growth as well as inflammatory responses, are meant to originate from release of toxic heavy metal ions [10–11] as well as from the formation of reactive oxygen species [5,12]. Thereby, nanoparticles are considered more hazardous than microparticles [13]. As to toxicity the field of reproduction biology is particularly interesting because the influence of nanoparticles on gametes is of great concern and has not been extensively studied up to date [14]. However, up to now toxicological studies concerning unintended release scenarios by nanoparticles have been impaired by the absence of adequate testing materials. Nanoparticles obtained from chemical synthesis routes are predominantly fabricated in the presence of artificial stabilizers. These stabilizers are known to interfere with toxicological assays [15], as reported, e.g., for cytotoxic CTAB [16–17] as well as for citrate [18–19], which is generally believed to be biocompatible. Next to toxicity, ligands like citrate may also influence particle properties, e.g., by inducing aggregation processes in the presence of biomolecules [20], and hence complicating bio-response studies. The removal of surfactants or residual ligands from colloidal nanoparticles is possible, e.g., by centrifugation [21], diafiltration [22] or tangential-flow filtration [23]. However, this process is very time consuming and often results in particle aggregation. Hence, pulsed laser ablation in liquids (PLAL) has proven to be a promising alternative for the synthesis of nanoparticles applicable in toxicity assays as it provides totally ligand-free colloidal nanoparticles [24–27]. This method is highly flexible concerning the target material which makes it particularly suitable for synthesis of alloy nanoparticles like, e.g., AuAg [28–29], NiFe and SmCo [30] and PtAu [31] nanoparticles, ablated directly from their corresponding bulk alloy targets. The particle size distribution of PLAL products is generally very broad [24,32]. Hence laser-fabricated ligand-free nanoparticles are excellent model systems to simulate implant wear processes and correlated toxic effects. Reference nanomaterials are particularly useful when their size and composition are controlled independently while their uniquely high purity is retained.

Hence, this article will highlight totally surfactant-free size control strategies for laser-fabricated nanoparticles and will comment on the stability of these particles in biological fluids. Additionally, laser-based synthesis methods for binary and ternary implant alloy nanoparticles are reviewed. The control of particle compositions is demonstrated with totally homogeneous AuAg alloy reference nanoparticle and examples are given as to how their composition is linked to nanotoxicologial effects.

## Review

### Size control of laser-generated nanoparticles without artificial ligands

In order to review nanoparticle size control strategies, gold nanoparticles were chosen as they are an excellent reference material for toxicological studies. Due to their exceptionally high stability concerning surface oxidation, gold nanoparticles do not release ions under physiological conditions. Hence, in contrast to gold atom clusters [33], gold nanoparticles are known to have a comparably low toxicity [34–35]. Hence, all adverse effects probably originate from the nanoscopic dimensions of the material, e.g., causing the formation of reactive oxygen species [33,36], and cannot originate from the material itself, like the ion release from nanoparticles composed of less noble materials.

**Figure 1:**
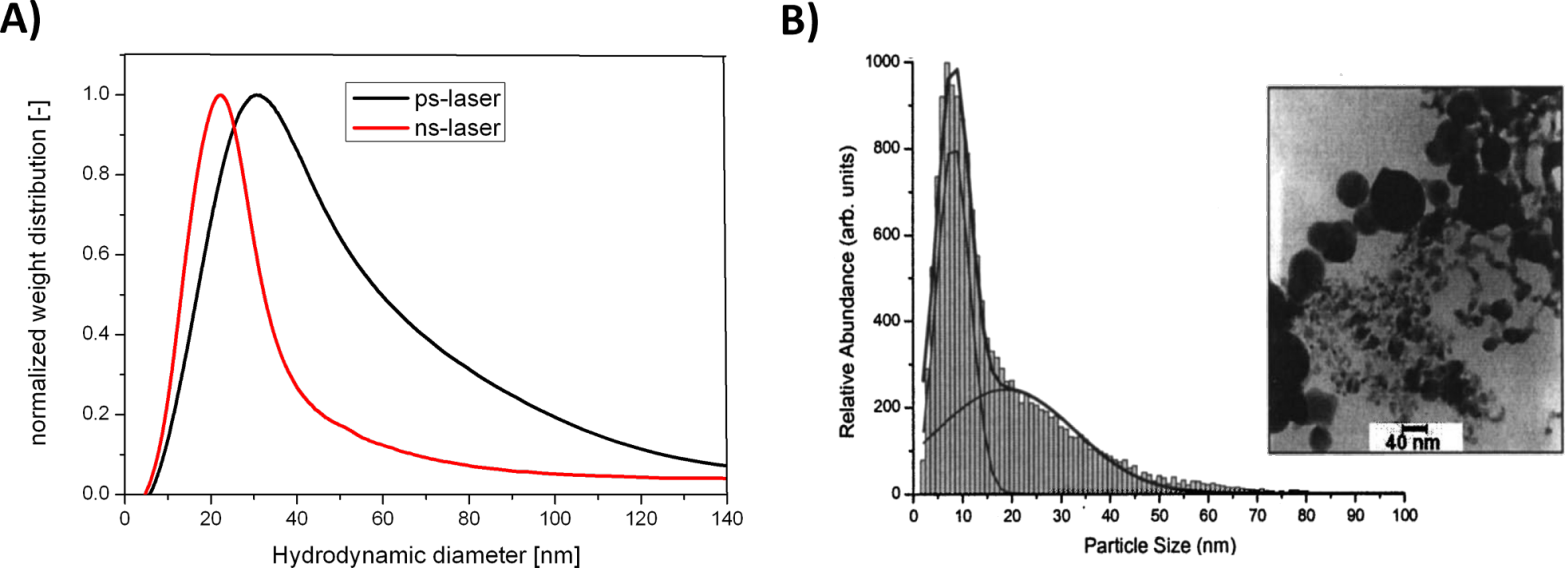
Influence of laser pulse length on particle size distribution. A) Representative normalized weight frequency of nanoparticle diameter of gold nanoparticles obtained from PLAL in deionized water using picosecond (black curve) and nanosecond (red curve) pulses. B) Gold nanoparticles obtained from femtosecond laser ablation showing a bimodal particle size distribution. (Reprinted with permission from [50]. Copyright 2003 AIP Publishing ICC).

The fabrication of gold nanoparticles by PLAL has been extensively examined in numerous studies, while ablation may be performed in aqueous media [37–39] as well as in organic solvents [40–41]. The obtained particles possess unique surface characteristics which are not reproducible by any other synthesis route. In that context, XPS measurements were used to verify that in aqueous media 3–6% of the surface atoms were oxidized to Au^+^ and Au^3+^ [42], while oxidation is most likely caused by dissolved oxygen or radical species formed during the ablation process. Surprisingly, similar studies, more recently conducted, revealed no elevated surface oxidation in laser-fabricated nanoparticles in pure water. This was attributed to the application of different laser pulse lengths but has not yet been sufficiently explained [43]. However, even though oxidation is meant to generate a positive surface charge, zeta potentials of laser-generated gold nanoparticles are all negative and titration with the positively charged ligand CTAB was used to confirm the presence of anionic surface moieties [42]. The most probable explanation is that positively charged gold surfaces attract water molecules, oxygen and carbon dioxide, forming a pH-dependent equilibrium of Au–OH/Au–O^−^ and Au–CO_3_^−^ groups [44–45]. Generally, these surface charges lead to a good colloidal stability due to electrostatic stabilization. An exemplary representation of typically broad size distributions of gold nanoparticles generated by picosecond and nanosecond laser ablation in deionized water, measured by analytical disk centrifugation, is shown in Figure 1A. These findings indicate that the polydispersity of the samples is generally larger when picosecond instead of nanosecond pulses are used during the ablation process. During PLAL a laser pulse hits the target and generates a plasma plume, whose onset is reported to occur tens of picoseconds after pulse absorption and contains a variety of different species like ionized atoms, clusters as well as larger fragments [24,46]. This is followed by the formation of a cavitation bubble on a microsecond scale, while the bubble confines crystalline nanoparticles [47]. In case of picosecond laser pulses the laser beam does not interact with the plasma plume while in the case of nanosecond pulses the plasma plume may absorb further energy from the laser pulse, which may be responsible for homogenization of the ejected material [24,48–49] and hence more narrow size distributions. In the case of femtosecond laser ablation, particularly at high laser fluence, alternative photomechanical ablation mechanisms like explosive boiling were reported, which result in bimodal particle size distributions (Figure 1B) [50].

Even though a relatively broad size distribution of nanoparticles fabricated by PLAL may be beneficial to simulate toxicological effects stemming from implant wear [6–8], it is a severe problem when a systematic study of nanoparticle toxicity needs to be carried out. Hence, the size of the nanoparticles needs to be precisely controlled over a wide range, while all artificial organic additives, potentially interfering with toxicity assays, have to be avoided. Here, the first idea would be to examine whether the laser ablation parameters themselves might be used to control particle size. To this end several authors examined the influence of laser wavelength [41,51], repetition rate [32] and fluence [50,52] on nanoparticle size distributions. Generally, a manipulation of the laser wavelength is only relevant in case it may be reabsorbed by the nanoparticles to induce photofragmentation. This reduces particle sizes and broadens size distributions, which was reported, e.g., during PLAL of gold with green (λ = 523 nm) and UV (λ = 355 nm) laser pulses [24]. An increase of the laser fluence generally triggers the formation of larger particles. However it also goes along with changes in particle productivity and may induce alternative ablation mechanisms causing polydisperse size distributions [27,50,53–54]. In general, variations of the laser parameters during synthesis alone are not suitable for the size control during ablation of bulk solids in liquid.

**Figure 2:**
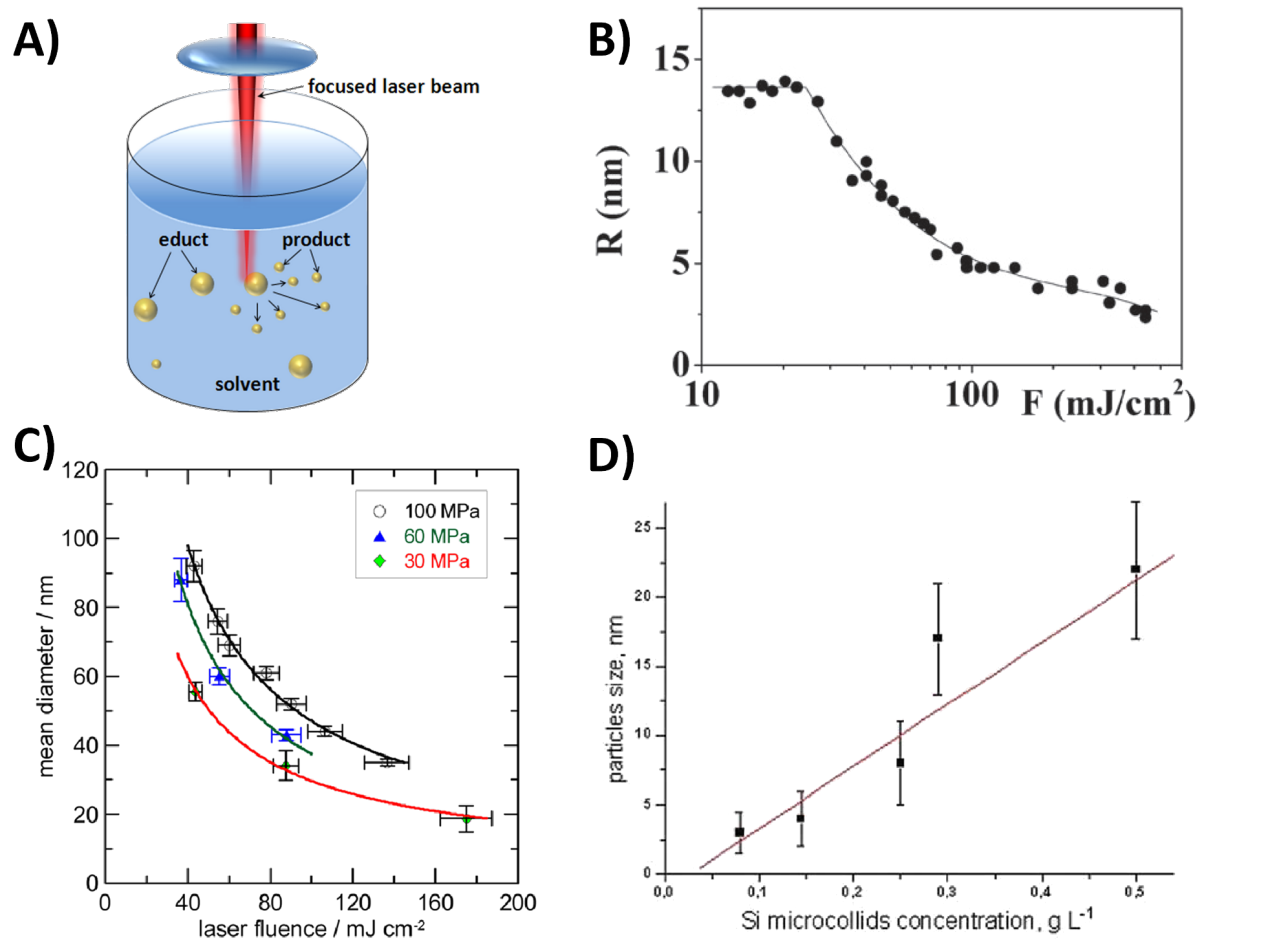
Size control of laser-fabricated nanoparticles by pulsed laser fragmentation in liquid (PLFL) tuning particles in a diameter range from 4–30 nm. A) Concept of PLFL. B) Size control by laser fluence in a size range from 2–15 nm (note that particle radius instead of diameter is plotted here) (Reproduced with permission from [68]. Copyright 2007 The Royal Society of Chemistry). C) Size control of gold nanoparticles by pressure (Reprinted with permission from [69]. Copyright 2013 American Chemical Society). D) Size control of Si nanoparticles by mass concentration during fragmentation of microcolloids. Reproduced with permission from [70]. Copyright 2013 The Royal Society of Chemistry).

The next possible strategy for size control may be ligand-free post-processing of the laser-fabricated nanoparticles. To this end a rather simple but nonetheless feasible approach is centrifugation at varying speed, yielding different size fractions [55]. This method was successfully applied in biological studies dealing with size dependent cellular uptake [56] and bioimaging [57] of gold nanoparticles. However, a rather labour-intensive preparation protocol as well as the problem of particle aggregation due to g-forces limits the use of this method. Another possibility for ligand-free size control of gold nanoparticles is re-irradiation with pulsed lasers, namely pulsed laser fragmentation in liquid (PLFL). Size control of gold nanoparticles by PLFL is induced by on- or off-resonance interactions of the nanoparticles with laser pulses [58] which eventually leads to a breakdown of nanoparticles and hence size reduction (Figure 2A). Basically, three mechanisms for PLFL in liquid have been postulated. The first one is a heating-melting-evaporation-mechanism, which basically implies that the onset of particle fragmentation occurs at the boiling point of the material and emission of smaller particles transpires layer by layer [59–60]. The second mechanism is Coulomb explosion, which occurs when large quantities of electrons are ejected from the particle due to laser-induced ionization, resulting in highly charged entities which eventually shatter due to charge repulsion [61–63]. A third mechanism, postulated for femtosecond pulses at very high fluence, is near-field ablation [64]. However, up to date it is unknown which mechanism prevails under certain experimental conditions and we refer to a review by Hashimoto et al. for further details [65]. Even though many groups used organic stabilizers such as citrate [59,66] or sodium dodecyl sulfate (SDS) [67], Amendola and Meneghetti [68] could show that size reduction might as well be exercised in totally ligand-free environments, where the application of nanosecond pulses at λ = 532 nm enables control of the particle diameters in a range from 4–30 nm for a variation of the laser fluence from 12–442 mJ/cm^2^ (Figure 2B). Furthermore, it was shown that PLFL may be influenced by the ambient pressure, though these effects were only shown for starting materials capped with ligands [69] (Figure 2C). Recent findings on Si nanoparticles also seem to indicate that during fragmentation of microparticles, particle concentration may be a determining factor influencing particle size [70] (Figure 2D). Hence PLFL seems to be a veritable method to vary particle sizes in gold nanoparticles, though this method primarily gives access to small particles and in some cases the parallel presence of educt and product during the process may lead to bimodal size distributions.

**Figure 3:**
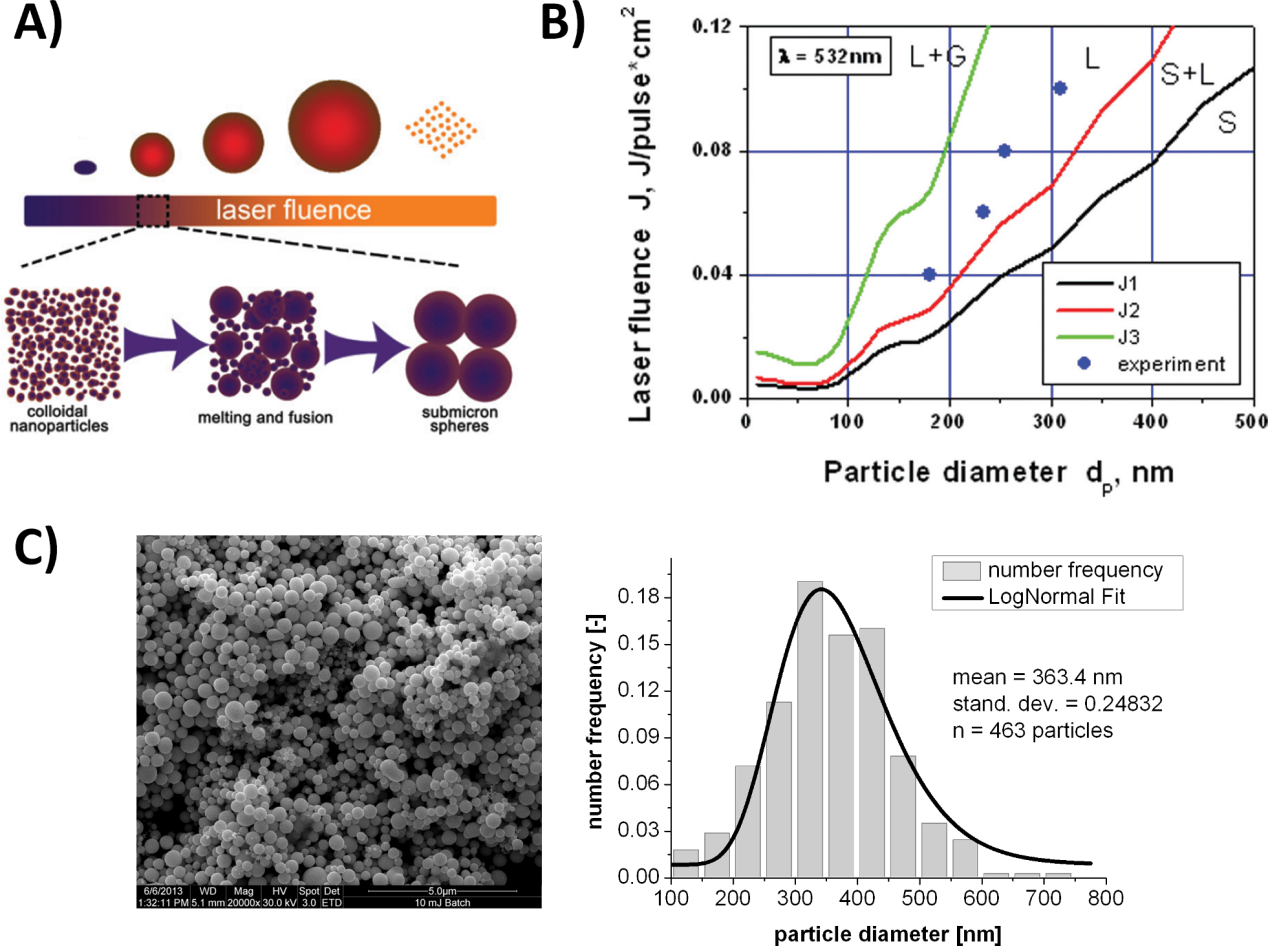
Size tuning of gold nanoparticles by pulsed laser melting in liquids (PLML) controlling particle diameters in a range of 100–400 nm. A) Heating-melting-evaporation mechanism for the formation of SMS. Higher laser fluence induces formation of larger particles until fragmentation occurs when a certain threshold energy is reached (Reproduced with permission from [72]. Copyright 2013 John Wiley and Sons). B) Correlation between laser fluence and particle diameter including theoretical calculations of phase transitions [72] as well as experimental data for citrate capped nanoparticles [74] (Reproduced with permission from [72]. Copyright 2013 John Wiley and Sons). C) Totally ligand-free Au-SMS generated by laser post irradiation of aggregated particles including representative SEM image (left) as well as particle number distribution calculated from SEM images fitted with a log-normal function (right) [80].

As PLFL primarily yields smaller nanoparticles, a second post-irradiation strategy, particularly suitable for the synthesis of larger particles is pulsed laser melting in liquid (PLML), which requires aggregated starting material [71] to be post-irradiated at moderate laser fluence. This process is reported to follow a heating-melting-evaporation-mechanism as stated by Pyatenko et al. [72]. When a nanoparticle aggregate in solution is irradiated by a nanosecond laser pulse exceeding a certain energy threshold, the structure melts and forms a liquid droplet. This melting process is highly localized in the vicinity of the particle due to high extinction cross sections of aggregated structures [73]. This heating process is followed by a rapid cooling upon decay of the laser pulse after 10^−6^–10^−4^ s, leading to solidified spherical nanoparticles (Figure 3A) [72]. Based on this mechanism the available particle size may be controlled by the pulse energy, as the melting point is size dependent and melting of larger particles requires higher energies. For gold, this concept is illustrated in Figure 3B [72] where the black curve (J1) depicts the onset of melting, the red curve (J2) indicates the point where all the material has melted, the green curve (J3) shows the evaporation threshold and blue dots mark experimental data [74]. This means 200 nm particles are predominantly formed at ≈40 mJ/cm^2^ while 300 nm particles are obtained at ≈75 mJ/cm^2^. Based on PLML, Amendola et al. [68] were able to increase the size of gold nanoparticles from ≈20 to ≈30 nm. However, PLML primarily yields particles in a submicrometer size range from 200–400 nm (sub-micrometer spheres, SMS), which could be obtained from multiple metals [75–76] and metal oxides [77–79]. In case of gold, SMS were primarily obtained from citrate-capped nanoparticles [74], while pyrolysis of the citrate induced aggregation is followed by the controlled formation of SMS. Unfortunately, this method may result in the formation of pyrolysis products from the degraded citrate [74], which may interfere with toxicity assays. Hence, inorganic salts (NaCl) may be used in order to induce nanoparticle aggregation [80], a strategy well established in literature and inspired by previous work on the impact of electrolytes on laser-fabricated gold nanoparticles [81]. The procedure to induce nanoparticle aggregation by electrolytes has been previously reported in context with PLML during the formation of ZnO-SMS [71]. However, the reader should note that in case of ZnO, biocompatibility of these SMS may be further compromised due to the possibility of elevated Zn^2+^ ion release upon laser irradiation. Zn^2+^ ions are known to have adverse effects on biological systems by chelating biomolecules and inducing toxic effects due to oxidative stress. These effects are reviewed elsewhere in more detail [82–83], while this paragraph further focuses on noble metals such as gold for which ion release is negligible. Biocompatible Au-SMS were synthesized in a cylindrical batch where laser-generated gold nanoparticles were exposed to NaCl at an ionic strength of 100 mM and consecutively irradiated with a nanosecond laser at 532 nm and 10 Hz repetition rate for 20 min at a fluence of 45 mJ/cm^2^. SMS were reproducibly obtained at diameters of 300–400 nm and an exemplary SEM images as well as a derived size distributions are shown in Figure 3C. As to the enormous potential of Au-SMS, e.g., in bioimaging, the toxicological potential of these materials needs to be assessed and PLML preceeded by electrolyte-originated aggregation offers an excellent opportunity to study these effects without side effects by surfactants or ligands.

Above, the presence of any organic stabilizers has been completely excluded during the size control strategies. However, organic ligands are present in all biological systems anyway so the application of additives strictly limited to this environment might be an elegant route for size control. Several groups could actually demonstrate that the presence of organic ligands during the PLAL-process (in situ conjugation) may be used for size control, predominantly yielding reduced particle sizes and narrowed size distributions [84–85]. This was also shown for biomolecules like oligonucleotides [52,86], peptides [87] and proteins [68], species native to biological systems and hence omnipresent in toxicity assays. However, in situ conjugation always entails the risk that nanoconjugates are destroyed by post irradiation [88–90], leading to pyrolysis products with unpredictable side effects. Even though this effect could be minimized by careful adjustment of laser parameters [91] and pH [89], these bioconjugates are not suitable for toxicity assays. Furthermore, it should be noted that laser-irradiated particles themselves, possess excited electronic states during the PLAL formation process. Certainly, particles in these transition states may unpredictably react with biomolecules due to electron transfer processes in case these biomolecules are present in situ. However, these excited states have a very short lifetime and primary particle formation is estimated to be finished within 10^−4^ s [27]. Hence, the resulting particles are not believed to possess additional adverse effects when added to biological samples ex situ.

**Figure 4:**
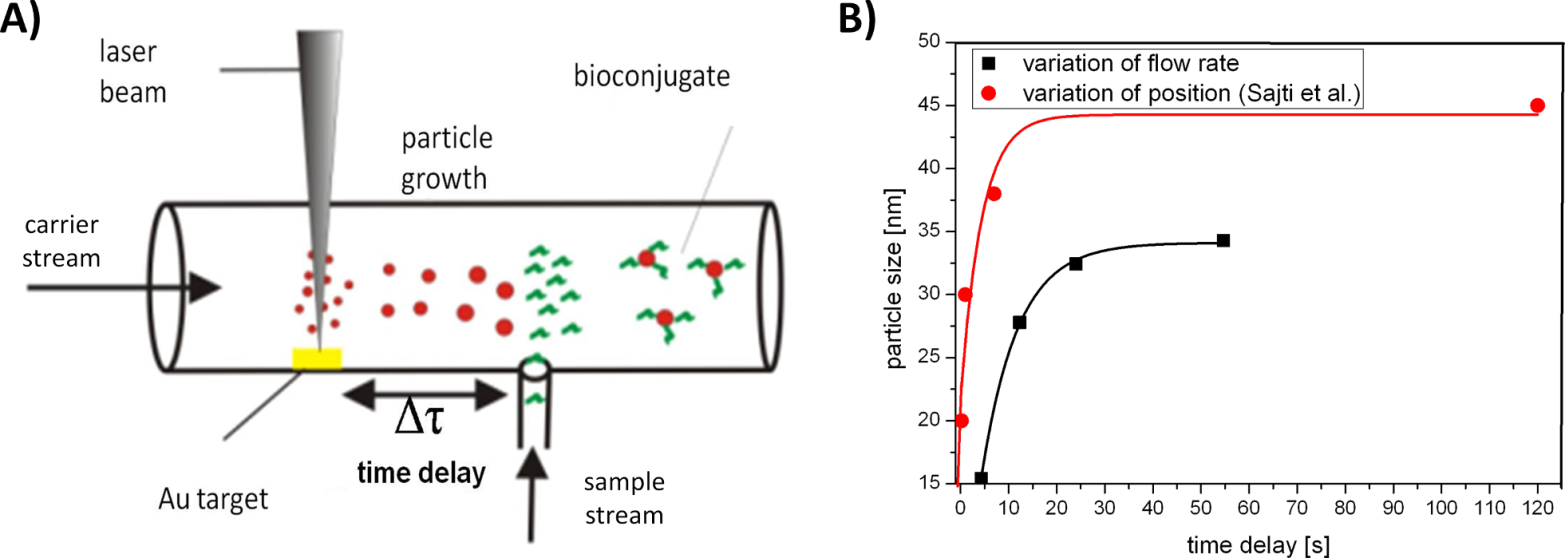
Size control by delayed conjugation in liquid flow allowing size control from 15–45 nm. A) Concept of delayed bioconjugation in liquid-flow. B) Size control by delayed conjugation by variation of flow-rate (black curve) and position of biomolecule injection (red curve) (data adapted from [92]).

Consequently, it is absolutely vital to geometrically separate the sites of ablation and bioconjugation, an approach possible in a flow-through reactor applying a process named delayed bioconjugation (fast ex situ bioconjugation) [92–93]. To this end laser ablation is carried out in a flow through reactor, while biomolecules are added at specified time delays. As gold nanoparticles generated in a carrier stream tend to grow on, possibly due to coalescence of small clusters milliseconds to seconds after synthesis [94], a specific quenching of that growth by biomolecules enables size control. This concept is illustrated in Figure 4A. Time delay during delayed bioconjugation may, for once, be controlled by the position of injection, which was demonstrated by Sajti et al. [92] where peptides were used as quenchers and particle diameter could be controlled in a range from 20–45 nm (Figure 4B, red curve). A complementary strategy involves variation of the residence time by flow rate in a range from 0.5–8 mL/min (corresponding to time delays of 4–54 s). In this case quenching of particle growth was achieved by albumin addition at a fixed position in a flow-through reactor (reactor design described elsewhere [81]) (Figure 4C, black curve). Here, particle diameters were determined by analytical disk centrifugation and size control in a regime of 15–34 nm was possible. As experimental setups in both studies greatly differed in productivity, flow rate and reactor dimensions a comparison of both results is difficult. However, the fact that smaller particles were generally obtained during the flow-rate variation approach might be attributed to lower particle concentrations.

**Figure 5:**
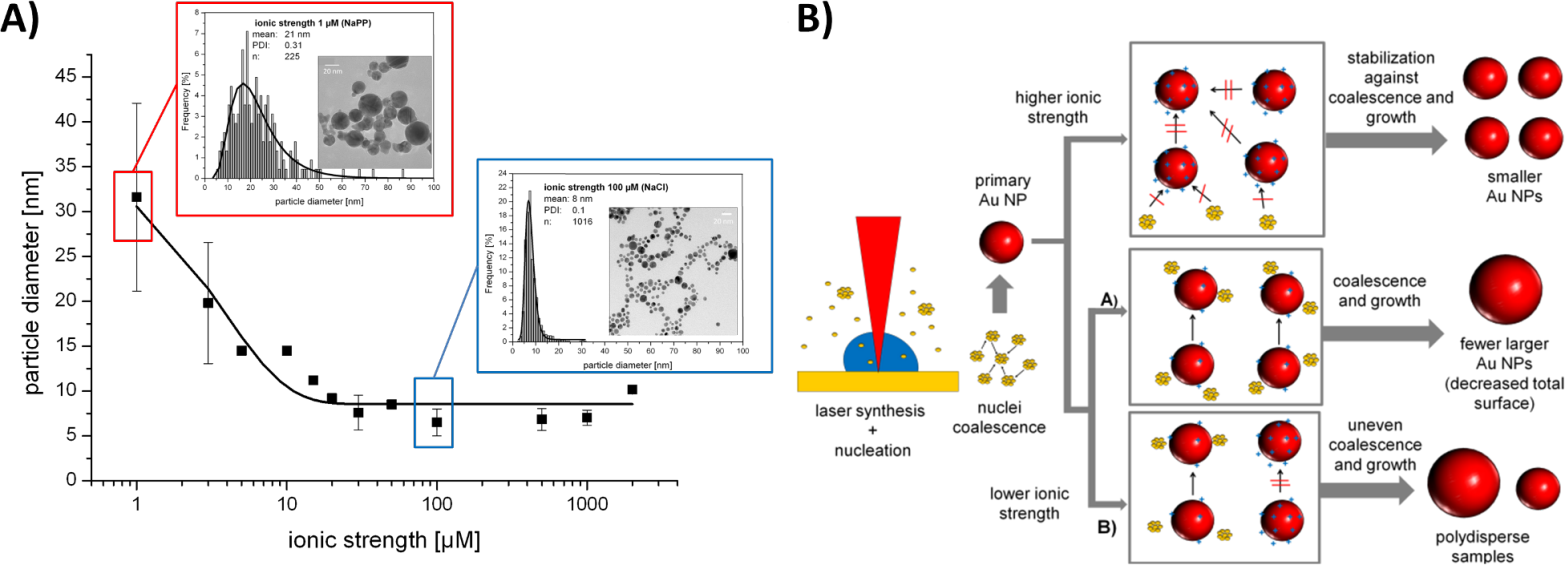
A) Size quenching effect of gold nanoparticles in the presence of different NaCl concentrations measured by analytical disk centrifugation as well as 2 representative size distributions and images from TEM (adapted from [81]). B) Proposed growth mechanism of laser-fabricated gold nanoparticles in the presence of electrolytes: Ions accumulate or adsorb on the surface of freshly formed primary nanoparticles stabilizing them electrostatically. At higher salinities growth is quenched at an early stage leading to smaller nanoparticles. At lower salinities, however, no sufficient stabilization is reached and particles growth continues and larger, polydisperse nanoparticles are found. (Reproduced with permission from [81]. Copyright 2013 The Royal Society of Chemistry).

An electrostatically-controlled approach for ligand-free size control of gold nanoparticle is the in situ addition of simple inorganic electrolytes like NaCl or sodium phosphate buffer. These additives are frequently found in most biological fluids and hence are not prone to interfere with toxicity assays. Even though the addition of ions is generally believed to compromise colloidal stability due to a reduction of the Debye length, previous studies reported an anion specific stabilization and destabilization of laser generated gold nanoparticles [44]. Furthermore, size control of these particles was reported depended on the salinity of the solution [24]. Recently, these effects were studied in a flow-through reactor and it was discovered that they already emerge in the Hückel regime at very low ionic strengths (1–200 µM). In this concentration range a size quenching effect occurred, leading to decreased mean particle diameters as well as reduced particle size distributions. This phenomenon was verified by TEM and analytical disk centrifugation confirming size control by electrolytes in a nanoparticle size regime from 6–30 nm (Figure 5A). Furthermore, it was elucidated that these effects are anion specific and do not occur in the presence of hard anions like F^−^ which destabilizes gold colloids even at low salinities.

This size quenching effect has been attributed to the adsorption of anions to primary particles during particle formation, which electrostatically stabilizes a defined surface area, inhibiting further growth and coalescence (Figure 5B) [81].

**Figure 6:**
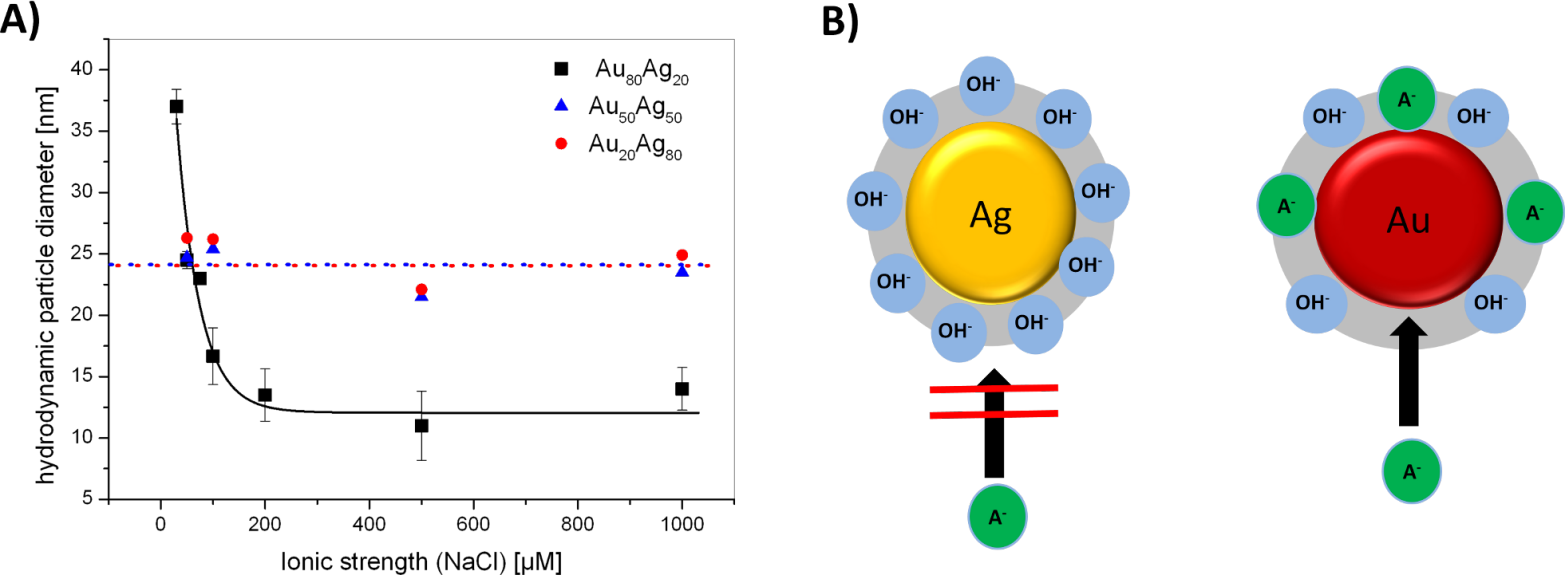
Size quenching of AuAg alloy nanoparticles is impaired by surface oxidation. A) Influence of in situ addition of NaCl on the formation of AuAg alloy nanoparticles at variable GMF. B) Cartoon illustrating elevated surface oxidation of silver nanoparticles followed by accumulation of surface hydroxides, which electrostatically blocks further anion adsorption and hence prevents size quenching.

Next to gold nanoparticles, size control of less noble metal nanoparticles like silver or AuAg alloys may also be relevant for toxicological assays, as particle size, which goes along with changes in curvature and surface area, is known to alter the dissolution behaviour of nanoparticles [95–96]. This may lead to elevated ion release and associated toxicity with decreasing particle diameters. Prior studies with silver nanoparticles seem to indicate that their stability and size distributions may also be altered by electrolytes [97] hence size quenching by ions may be a suitable way. To this end AuAg alloy nanoparticles with gold molar fractions (GMF) of 0.2, 0.5 and 0.8 were fabricated in the presence of NaCl at salinities varying from 50 to 1000 µM. The hydrodynamic diameter of these particles was determined by analytical disk centrifugation. Interestingly, a size quenching effect, similar to gold, was only observed at a GMF of 0.8 for which particle size decreased from 37 nm to 12 nm with increasing ionic strength. However, for the GMF of 0.2 and 0.5 the hydrodynamic diameter remained constant at 25 nm over the entire concentration range (Figure 6A). As previously discussed, size quenching by ions is most likely attributed to specific anion adsorption on uncharged, hydrophobic metal surfaces, leading to electrostatic particle stabilization [81]. However, silver surfaces are characterized by a significantly higher surface oxidation compared to gold. This surface oxidation goes along with a higher portion of surface oxides and hydroxides, which, dependent on the pH of the solution, are generally negatively charged. Hence they electrostatically block adsorption of anions and prevent size quenching by additional ions like Cl^−^. This concept is illustrated in Figure 6B.

**Figure 7:**
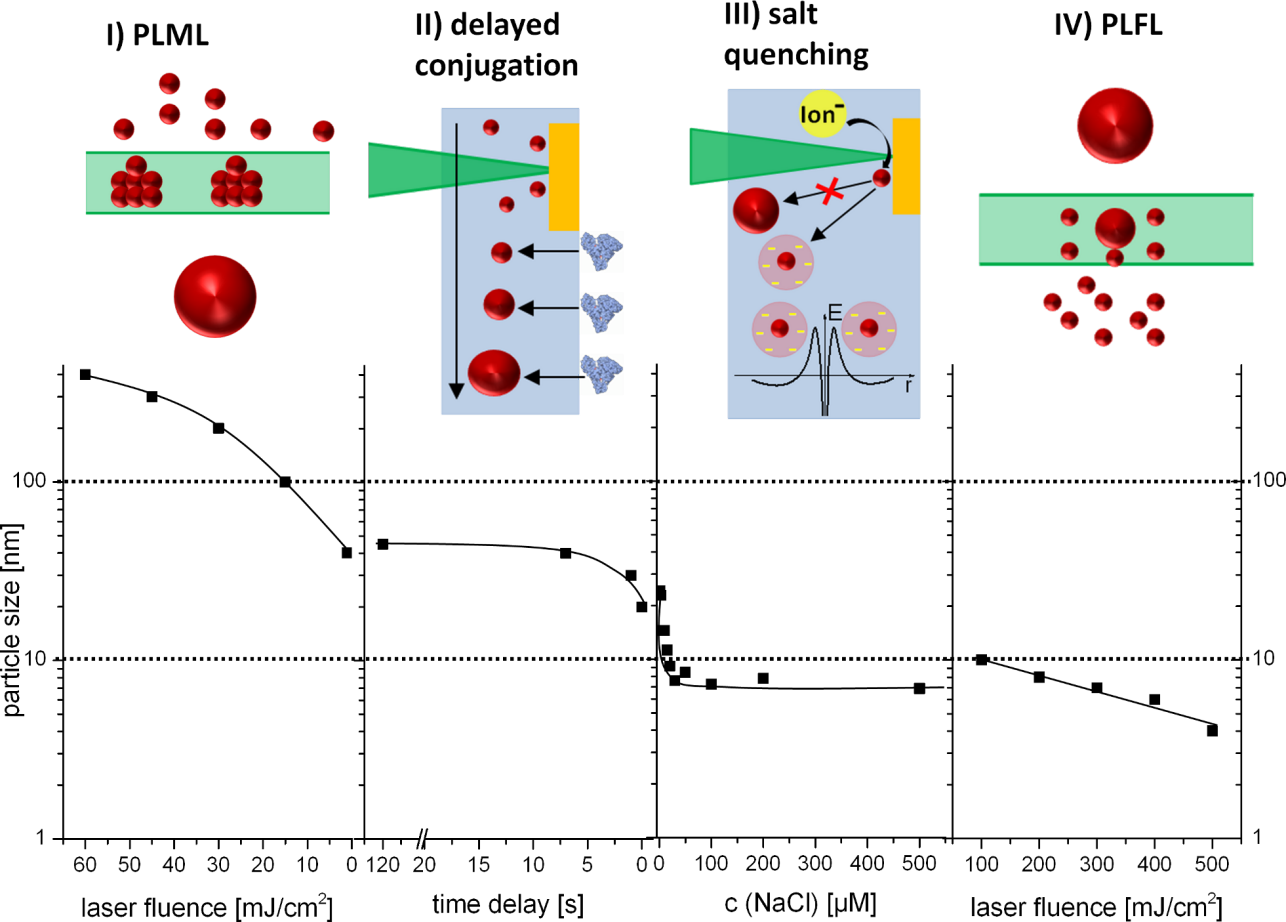
Size control for biocompatible gold nanoparticles by 4 different methods: I) Pulsed laser melting in liquid (PLML); II) Delayed conjugation; III) Size quenching by salts; IV) Pulsed laser fragmentation in liquid (PLFL).

In this paragraph we reviewed that size control of ligand-free gold nanoparticles may be basically performed by four different strategies including 1) pulsed laser melting in liquid, 2) delayed bioconjugation in liquid-flow, 3) addition of low salinity electrolytes and 4) pulsed laser fragmentation in liquid. Each of these methods enables size control in complementary regimes (Figure 7), covering an impressive particle diameter range of 4–400 nm. Furthermore, centrifugation of nanoparticle fractions may be a versatile alternative for particle size separation, although labour intensive protocols and particle aggregation limit its usefulness. Additionally, the concept of size control by electrolytes is transferable to alloys with a high GMF, though the effect seems to be overshadowed by surface oxidation.

### Fundamental aspects on the stability of ligand-free nanoparticles in saline biological media

In order to apply totally ligand-free nanoparticle reference materials in toxicity assays in biological media their stability and aggregation tendencies in these environments need to be understood. This is of paramount importance as it was shown that aggregates may have significantly different toxicological effects than single particles [98–99]. Hence this chapter is meant to elucidate fundamental effects concerning nanoparticle stability, neglecting the specific influence of surface charge, which was fundamentally addressed in the previous paragraph and in other recent publications [45,100]. Furthermore details concerning surface charge–cell interactions, which are known to be involved in toxicity of nanoparticles are beyond the scope of this article and were described elsewhere [101–102].

Due to the absence of surface ligands, reference nanoparticles may be considered ideal hard spheres whose colloidal stability is entirely driven by electrostatic effects, which may be well characterized by their electrophoretic mobility (zeta-potential) [103]. Under these conditions, colloidal stability is basically influenced by three parameters including temperature, ionic strength and particle concentration. The effect of temperature can be evaluated considering that colloidal stability of nanoparticles in pure water is sufficient when the zeta-potential (ξ) exceeds the thermal energy of the particles [103]:

[1]
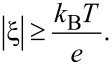


with *k*_B_ = 1.380 × 10^−23^ J/K being the Boltzmann constant and *e* = 1.602 × 10^−19^ C being the elemental charge. This assumption is derived from the frequently used dimensionless zeta potential [45,104–105], which equals 1 when thermal energy and zeta potential possess equal dimensions [45]. This assumption is valid as it has long been known that during measurements of the electrokinetic potentials in aqueous solutions the temperature dependence of conductivity, dielectric constant and viscosity are negligible up to a temperature of 343 K [106]. Based on these simplified assumptions, at room temperature (*T* = 293 K) a zeta-potential of ξ = 26 mV is required for stabilization while for biological assays, predominantly conducted at *T* = 310 K, slightly higher values of ξ = 27 mV are needed. Even though the effect of temperature seems relatively low it has to be considered, particularly when working with particles close to this stability threshold.

Additionally, the electrolyte concentration has a pronounced impact on nanoparticle stability as ions are known to screen surface charges in the electrical double layer, increasing the Debye parameter (κ). κ is reciprocal to the Debye screening length which describes the range of electrostatic repulsion forces that keep particles apart. It may be correlated to the ionic strength (*I*) by [107]:

[2]
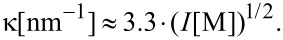


During ligand-free synthesis of nanoparticles ionic strengths are generally kept relatively low (≈100 µM) in order to control the particle diameter and to ensure colloidal stability. Such salinities, however, are not realistic for toxicological assays and are solely required during synthesis. Hence, upon exposition to biological fluids like blood, significantly higher ionic strengths (≈200 mM) may be found. This means that upon addition of the nanoparticles to biological fluids, 1/κ is significantly reduced from 30 nm at 100 µM to 0.7 nm at 200 mM, indicating that electrostatic stabilization is completely lost under these conditions and spontaneous aggregation will occur. Note that even though these ligand-free nanoparticles are exceptionally susceptible to electrostatic influence, their unique electrostatically controlled nano-environment allows observations of gold nanoparticle buffering effects and specific ion adsorption which cannot be studied in ligand-stabilized systems [45]. Furthermore, it should be noted that the Debeye parameter is known to be temperature-dependent as well (κ ~ (1/*T*)^1/2^) [108] and the Debeye screening length (κ^−1^) increases with temperature, a correlation which needs to be considered when temperature and ion effects are meant to be simultaneously evaluated in great detail.

**Figure 8:**
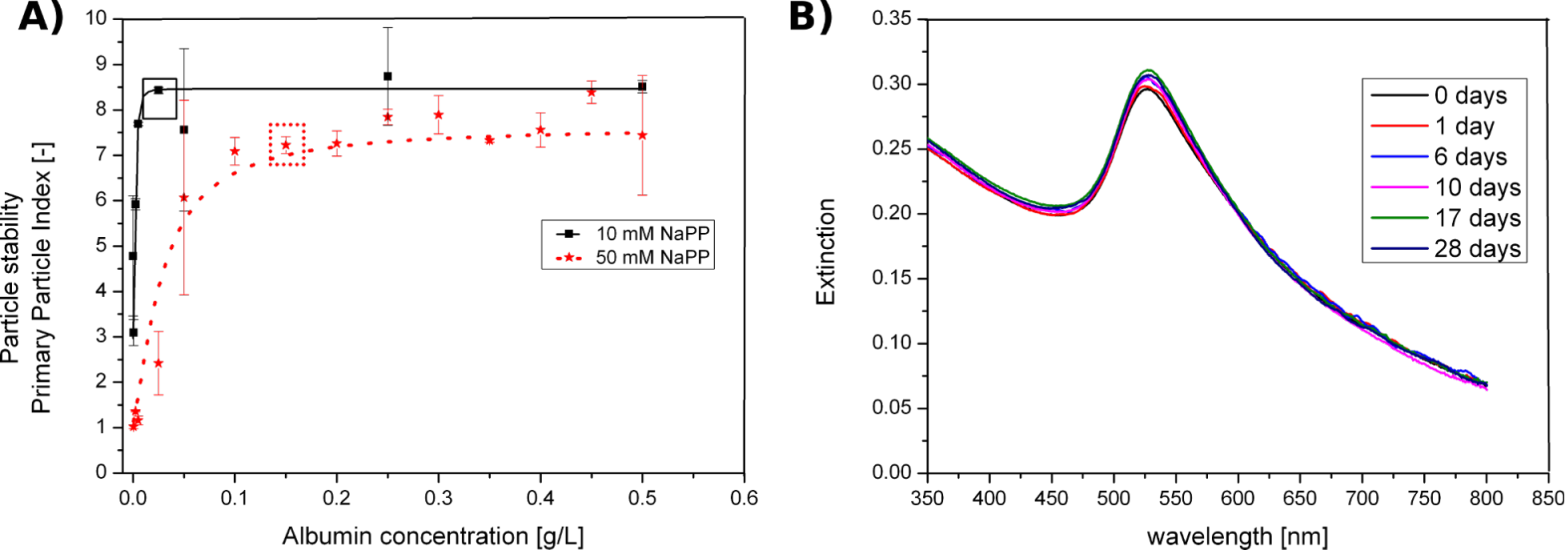
Stabilization of gold nanoparticles in the presence of serum albumin. A) Colloidal stability of gold nanoparticles at varying albumin concentrations and different salinities of sodium phosphate buffer (NaPP). The boxes indicate the minimum stabilizing concentrations (Reproduced with permission from [81]. Copyright 2013 The Royal Society of Chemistry). B) Long-term stability of gold nanoparticles in serum-rich Androhep-medium for 28 days. (Reproduced with permission from [81]. Copyright 2013 The Royal Society of Chemistry).

Another indirect influence on colloidal stability is given by the particle concentration. Dilution of nanoparticles, expected to occur when nanoparticles are added to a biological medium, will lead to a higher interparticle distance. This makes particle collisions, which are required to induce aggregation processes following second order kinetics [109], less likely and colloidal stability will increase upon dilution. Based on the above mentioned three determining factors it may be concluded that ligand-free colloids may only be used as primary particles in biological assays when they are highly diluted, while at higher concentrations mainly aggregates have to be considered. However, the fate of nanoparticles in biological fluids is not only dictated by electrostatic effects. Nanoparticles are known to spontaneously react with organic medium components, predominantly serum proteins, which rapidly (<0.5 min) form a stable protein corona on the nanoparticles [110], known to stabilize the particles against aggregation by sterical effects [111]. The mechanism of protein corona formation is still under vivid debate, though it is generally believed that proteins with high affinities strongly bind to the nanoparticle surface forming a hard corona, while other proteins more loosely bound form a soft corona. The corona is reported to be highly dynamic. Depending on the medium more abundant proteins are quickly adsorbed but gradually replaced by proteins with higher affinity [112–113]. However, more recent studies involving the time-resolved screening of 300 blood proteins by proteomics seems to indicate that the time resolved changes in the composition of the corona are not that pronounced [110]. However, protein-nanoparticle interactions for totally ligand-free nanoparticles have not been extensively studied [68], though it is reported that protein deformation is more likely to occur on bare nanoparticle surfaces [111]. In order to examine the influence of protein stabilization on ligand-free nanoparticles, albumin may be an appropriate model substance, which is known to be abundant in the protein corona and is one of the most frequent proteins in serum-containing cell culture media [114]. It was recently shown that ligand-free laser-fabricated gold nanoparticles could be successfully stabilized by albumin [81]. Additionally, the required albumin concentration is dependent on the ionic strength of the medium (Figure 8A), most likely caused by the interplay between two opposing forces, namely steric stabilization by albumin and destabilization by screening of surface charges at higher salinities. In this context aggregation tendencies evaluated using UV–vis spectroscopy, allow to compare the portion of gold interband absorbance (380 nm) to the scattering of aggregates in the NIR regime (800 nm) estimated by the primary particle index [81]. Consecutively, ligand-free gold nanoparticles, which were exposed to a full serum-containing medium, were stable for a period of 28 days as albumin concentrations in that medium exceed minimum stabilizing concentrations by a factor of 10 (Figure 8B) [81]. Hence the abundance of proteins in all biological systems and their stabilizing effect on nanoparticles allows application of unaggregated nanoparticles in toxicological assays. Here, sterical stabilization by proteins generally overshadows electrolyte-induced destabilization and allows utilization of highly concentrated colloidal nanoparticle reference materials.

### Laser-fabricated binary and ternary implant alloy nanoparticles

In the previous chapters size control and stability of relatively inert reference materials were reviewed. However, for toxicity assays nanoparticles from implant alloy are much more relevant. Hence, this chapter reviews laser-based synthesis of these materials, concentrating on NiTi and Cr-steel as examples for medically relevant binary and ternary alloys.

**Figure 9:**
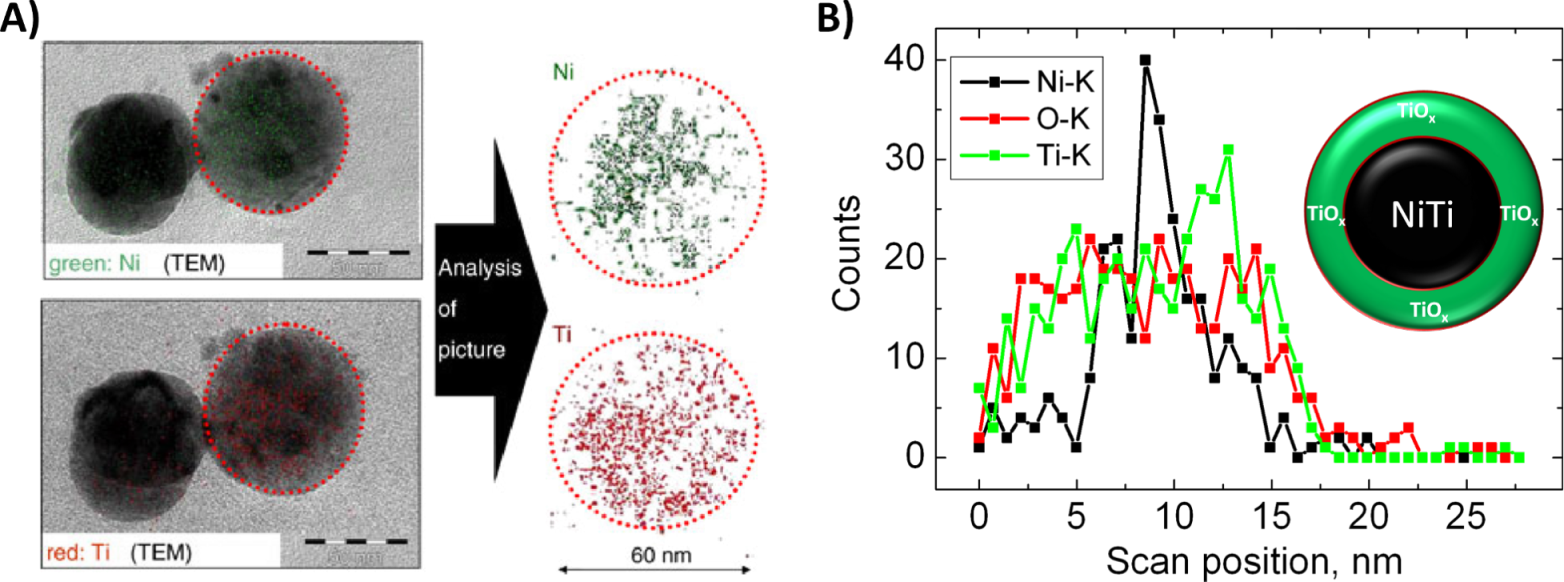
A) Stoichiometry of NiTi nanoparticles. Left: TEM images of NiTi nanoparticles generated by femtosecond laser ablation in acetone (transferred into polymeric matrix and cut by an ultramicrotome). Right: electron energy loss spectroscopy mapping of NiTi nanoparticle with 60 nm diameter. Green spots mark coordinates of nickel, titanium is marked red (Reproduced from Figure 4 in [115] with kind permission. Copyright 2010 Springer Science and Buisiness Media). B) TEM-EDX-line scan of a representative NiTi nanoparticle, laser-fabricated in water, indicating a segregated Ni-TiO*_x_* core–shell structure as illustrated in the cartoon.

NiTi alloys are widely applied in medicine due to their outstanding mechanical properties such as superelasticity and shape memory effect, which was reported to occur even on a nanoscopic scale [115]. These characteristics make NiTi alloys particularly suitable, e.g., as stent material [116–118] and scaffolds in bone tissue engineering [119]. Synthesis of NiTi nanoparticles by laser ablation in liquid has been described in the literature [115,120] and these nanoparticles were frequently adsorbed to implant surfaces as nanocoatings [121]. Even though Ni nanoparticles, and particularly the Ni^2+^ ions which they release, are known to have an immunogenic effect [122] and are reported to be cytotoxic [123–124], NiTi alloy nanoparticles tend to show no significant adverse effects. This was reported for endothelial and smooth muscle cells where nanoparticle concentrations of less than 10 µM were non-toxic [11]. On the contrary, NiTi coatings are even reported to improve the cytocompatibility of implants due to their surface texture [125]. As titania nanoparticles are generally considered to possess a relatively low toxicity [126–127], possible adverse effects of NiTi alloys are prone to originate from nickel. So in order to evaluate toxic effects from these particles, TEM-EDX and EELS (electron energy loss spectroscopy) were used to analyze the ultrastructure of the particle by localizing nickel on a single-particle basis. Single particle EELS of a NiTi particle, laser-fabricated in acetone and embedded in a polymer, revealed a totally homogeneous ultrastructure [115] (Figure 9A). In contrast, NiTi nanoparticles fabricated in pure water and analyzed by TEM-EDX including line scans of ten different nanoparticles showed highly polydisperse elemental compositions. Here Ni-rich (Ti: 16 ± 3%; Ni: 84 ± 3%) and Ti-rich (Ti: 79 ± 9%; Ni: 21 ± 8%) particles as well as particles with evenly distributed elemental compositions (Ti: 50 ± 4%; Ni: 50 ± 4%) were found. Interestingly, particles with even distributions as well as Ti-rich particles revealed a highly distinctive elemental segregation (Figure 9B); the predominant structure in this case was a Ni core surrounded by a TiO*_x_* shell. This titania shell could inhibit Ni diffusion to the NP surface and might be responsible for a low Ni^2+^ ion release, which increases the biocompatibility of this material. Deviations in findings between NiTi nanoparticles synthesized in acetone in the presence of polymers and in water might be due to surface oxidation which is believed to be far more pronounced in an aqueous medium. Consequently, elemental segregation in NiTi nanoparticles requires a certain degree of surface oxidation, which is likely to occur anyway during unintended nanoparticle release scenarios in biological systems.

**Figure 10:**
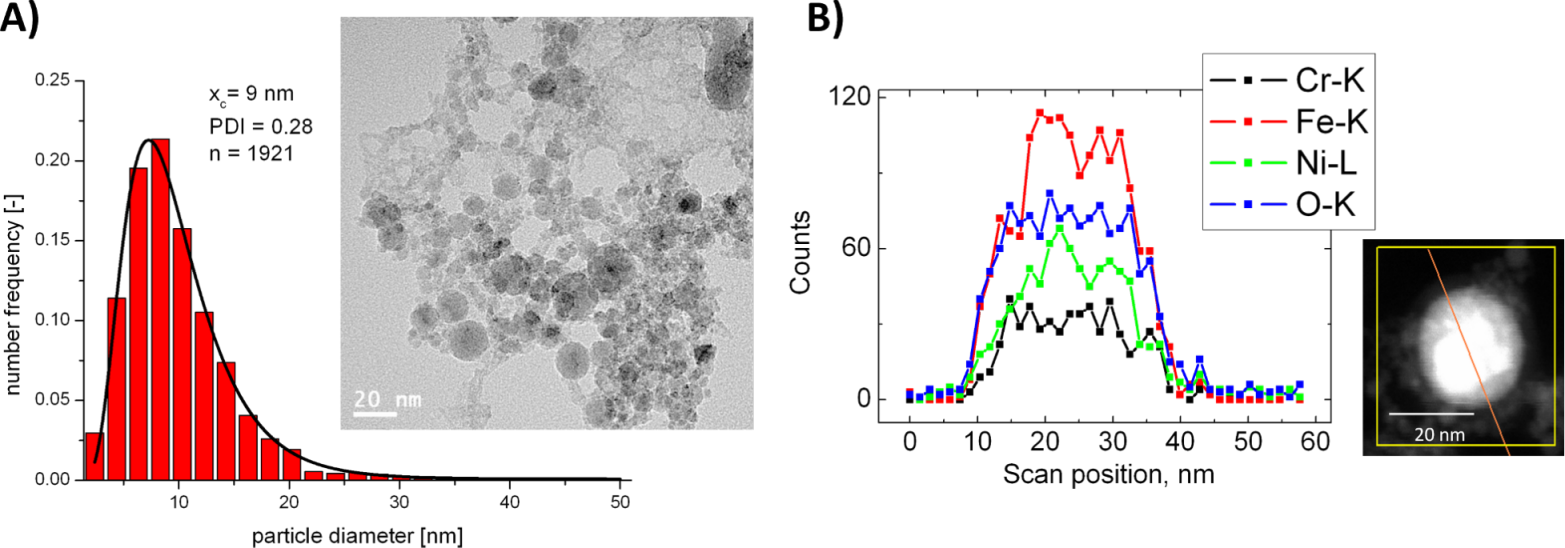
Nanoparticle fabrication from medically applied ternary stainless steel bulk material (material reference number 1.4404) yields particles with totally homogeneous elemental distribution. A) TEM size distribution of ternary steel alloy nanoparticles. B) Representative EDX line scan of a single steel nanoparticle revealing a totally homogeneous elemental distribution.

Next to binary alloys like NiTi, nanoparticle release and related toxicity from alloys with more complex elemental compositions like stainless steels is particularly interesting as it is found in a broad range of medical devices and implants [128–129]. Synthesis of steel nanoparticles by PLAL has not been addressed in the literature, though the fabrication of FeO*_x_* [130–132] as well as NiFe [30] and FePt [133] alloys nanoparticles has been reported. Recently, PLAL was applied to fabricate nanoparticles from austentic stainless steel samples in water, with bulk target compositions Fe 65–75%, Cr 15–20%, Ni 10–15% (unpublished work). Nanoparticles obtained from these ternary alloys could be stabilized in biological media and particle size distributions determined by TEM revealed a broad distribution of particle diameters (Figure 10A). Single particle EDX and EDX line scans of ten different particles revealed an elemental composition of Fe 65 ± 9%, Cr 17 ± 7% and Ni 18 ± 9%, which is in good correlation with the composition of the bulk target. The EDX line scans revealed significant surface oxidation, basically following the iron content. However, the elemental distribution in all particles was completely homogeneous (Figure 10B), even though an alloy of three metals with highly diverse chemical properties was used. Hence PLAL gives access to ternary alloy nanoparticles with totally homogeneous elemental compositions which is not possible by any other synthesis route. Furthermore, these findings seem to indicate that elemental segregation in laser fabricated alloy nanoparticles may not be solely dominated by surface oxidation but also by the elemental composition of the alloy particle. In order to further examine this phenomenon, alloy nanoparticles with well-defined elemental compositions are required, whose synthesis is reviewed in the next chapter.

### Laser-fabricated alloy nanoparticles with controlled composition

In order to synthesize alloy nanoparticles with controlled elemental compositions AuAg proves to be an ideal model system as the phase diagram of gold and silver is relatively simple and the elements form isotypic crystals. Additionally, the system AuAg is a suitable model system to study toxicity as it consist of gold, known to be biocompatible [34], as well as silver. The latter is frequently reported to be harmful to biological systems where it is often used due to its antimicrobial effect [134–136].

**Figure 11:**
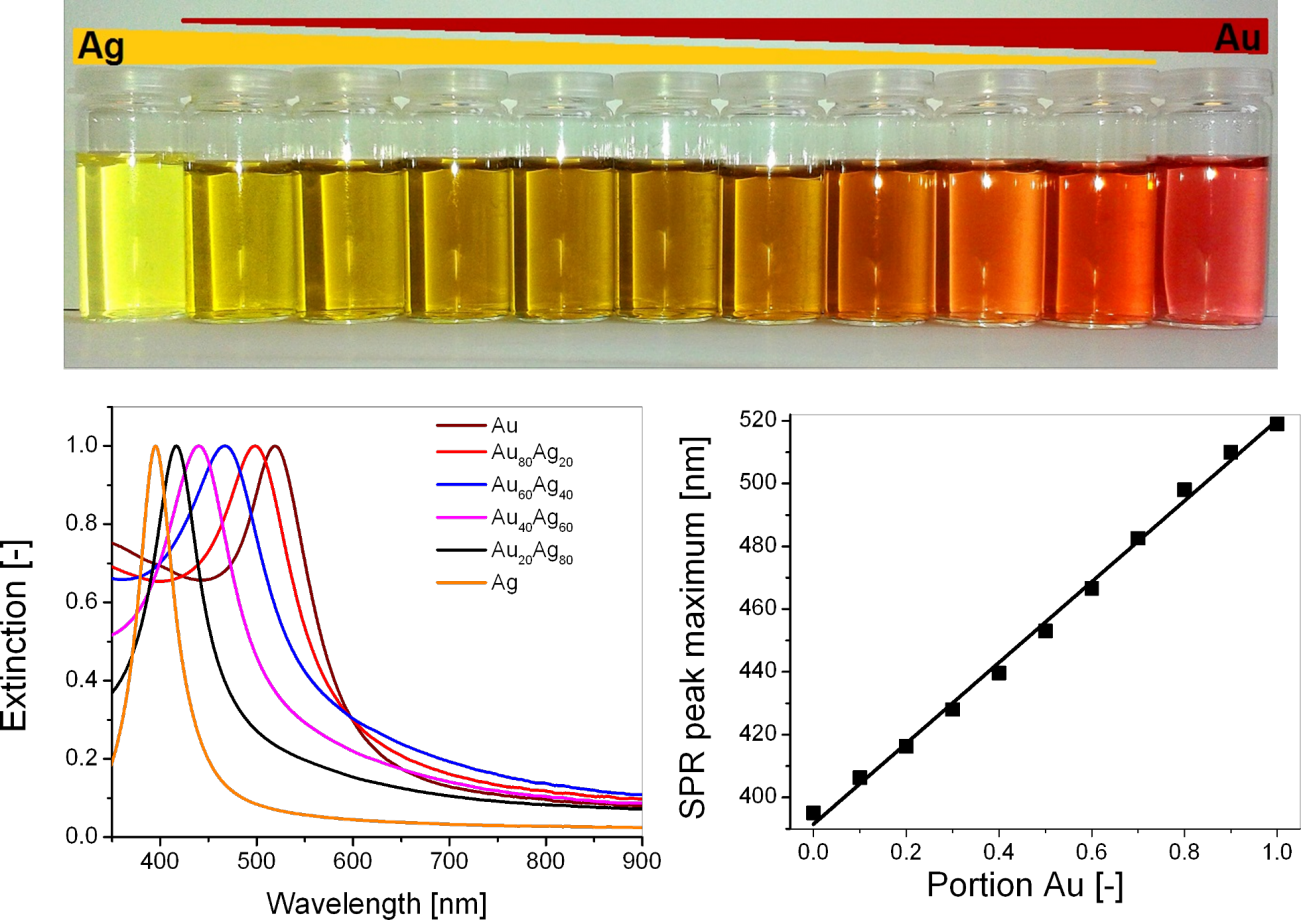
Precise tuning of particle composition in AuAg alloy nanoparticles. Top: Representative colloids of variable composition. Bottom left: Representative UV–vis spectra. The occurence of a single peak indicates alloy formation. The position of the SPR-peak red shifts with increasing portion of Au (GMF). Bottom right: SPR-peak position linearly shifts with increasing GMF. (Reproduced with permission from [35]. Copyright 2014 The Royal Society of Chemistry).

Next to the abundance of potentially toxic stabilizers, the synthesis of AuAg alloy nanoparticles by chemical methods proofs to be difficult as parallel co-reductions of Au^3+^ and Ag^+^ metal salts operates at different rates due to different redox potentials. This often favors element segregation and the formation of core shell structures in the resulting nanoparticles [137–138]. Laser-based synthesis methods have proven to be a veritable alternative to generate AuAg alloy nanoparticles with homogeneous elemental distributions even on a single particle scale [29], which were verified by UV–vis spectroscopy as well as TEM-EDX [28–29,139]. In the UV–vis spectrum, laser-fabricated AuAg alloy nanoparticles only show one SPR extinction maximum [140–141], which linearly shifts with the elemental composition [28–29,35,139] as illustrated in Figure 11. Theoretical modelling indicates deviations from a linear correlation for silver rich particles [142].

**Figure 12:**
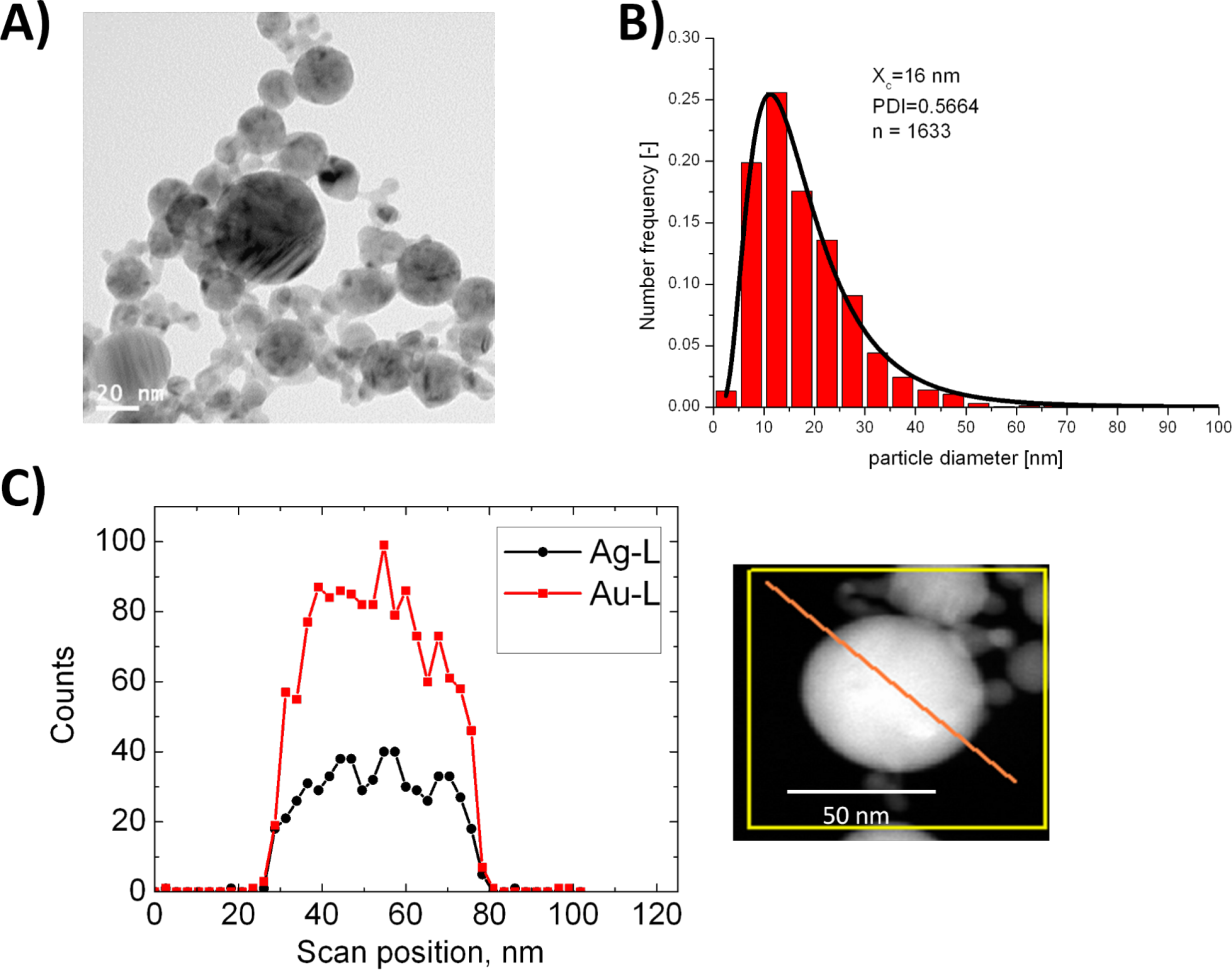
Laser-fabricated AuAg alloy nanoparticles possess a completely homogeneous elemental distribution. A) Representative TEM image acquired at GMF = 0.5. B) Particle size distribution of AuAg alloy nanoparticles at GMF = 0.5. C) EDX line scan at GMF = 0.8 indicating totally homogenous elemental distribution on a single particle level.

Laser-based synthesis of AuAg alloy nanoparticles may be conducted by two different methods both avoiding chemical precursors and stabilizing ligands. The first one is based on laser post irradiation of colloidal mixtures inducing an alloying process [29,143–144], which was reported to occur via core-shell intermediates [145]. Another approach entails ablation of silver targets in the presence of gold nanoparticles [146]. Post-irradiation method proofs to be highly flexible as to the composition of the resulting nanoparticles. However they are limited to femtosecond lasers, have a comparably low productivity, and requires a two-step process. The second strategy involves direct PLAL from the corresponding alloy targets [28,139] an approach adapted to fabricate AuAg alloy nanoparticles in a flow-through setup (described elsewhere [81]) using targets with gold molar fractions (GMF) of 0.2, 0.3, 0.4, 0.5, 0.6 and 0.8 (surface composition accuracy ±1%). This allowed particle generation at extraordinarily high mass concentrations (>300 µg/mL). The particles were analyzed by HR-TEM as well as single-particle EDX and EDX line scans of ten individual particles. The results are summarized in Table 1 while an exemplary image and the size distribution for GMF = 0.5 as well as a line scans for samples with GMF = 0.8 are shown in Figure 12. These findings indicate that alloy nanoparticles with broad size distributions are obtained, while the elemental composition of the target is retained on a single particle level. Furthermore, the line scans clearly indicate that the elemental distribution is totally homogeneous in the alloy nanoparticles.

**Table 1:** Summary of TEM/EDX results for AuAg alloy nanoparticles for different gold molar fractions (GMF). Errors in the mean particle diameters originate from the standard deviation of the log-normal fitting curves, while errors in the elemental compositions were determined from the standard deviations of ten separately measured single particles.

Gold molar fraction (GMF) of bulk target	Mean particle diameter [nm]	Elemental composition Au:Ag of single nanoparticles (EDX)

0.2	13 ± 8	25:75 (±3)
0.3	13 ± 8	38:62 (±6)
0.4	11 ± 9	42:58 (±6)
0.5	16 ± 9	48:52 (±3)
0.6	10 ± 5	64:36 (±5)
0.8	15 + 14/ −7	82:18 (±3)

**Figure 13:**
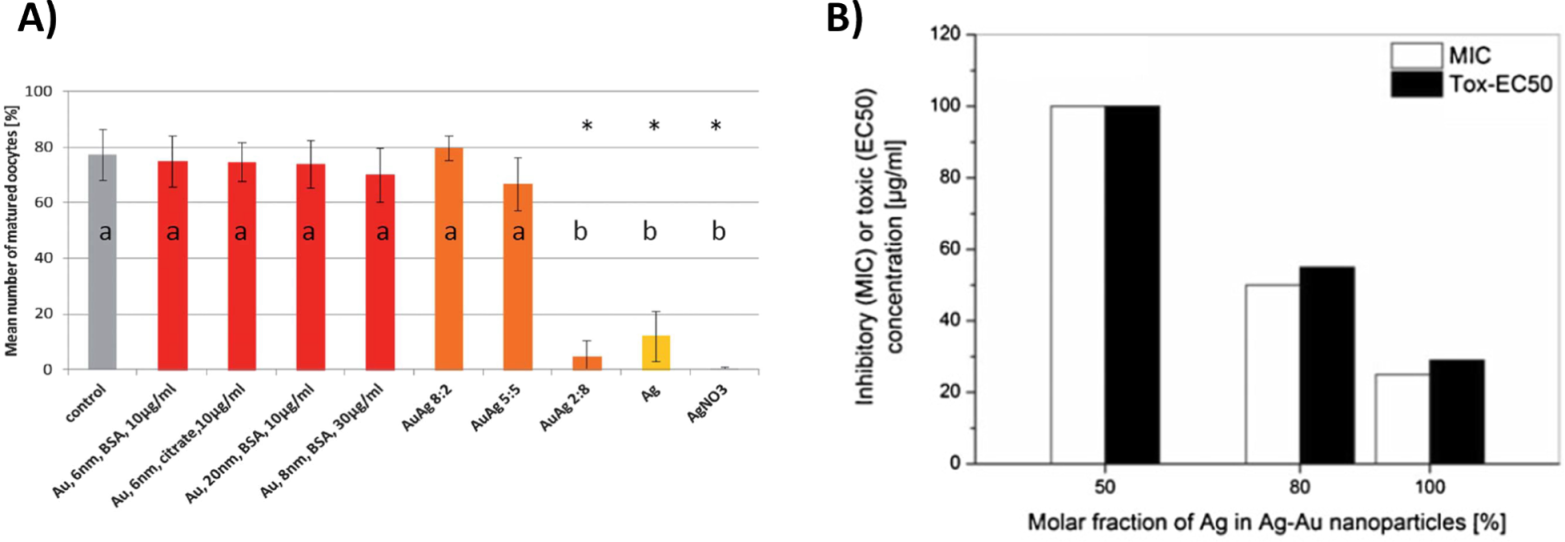
Bio-response of AuAg alloy nanoparticles is non-linearly correlated with the particle composition. A) Influence of AuAg alloy nanoparticles on oocyte maturation indicating a significant decrease at GMF = 0.2. (Reproduced with permission from [35]. Copyright 2014 The Royal Society of Chemistry). B) Inhibitory and toxic concentrations of AuAg alloy nanoparticle on viability of human gingival fibroblasts for varying silver molar fraction (adapted from [148]).

As totally homogeneous AuAg alloy nanoparticles with tunable elemental compositions on a single particle level could be synthesized it is highly relevant to link their elemental compositions to the toxicity of these materials and compare it to the pure gold and silver counterparts. Here, Tiedemann et al. [35] could show that oocyte maturation was unaffected when conducted in the presence of gold nanoparticles of variable size (6 nm and 20 nm), dose (up to 9 × 10^10^ particles/oocyte) and surface functionalization (BSA and citrate) (Figure 13A). In accordance with these findings spermatozoa exposed to gold nanoparticles in an albumin rich medium showed no functional impairment. However, previous examination in this context revealed decreased motility of spermatozoa incubated with gold nanoparticles in serum-free medium. These results were associated with membrane attachment of aggregated nanoparticles blocking surface thiol groups involved in sperm movement [147], which probably does not occur when the nanoparticles are coated by an albumin corona. In contrast, silver nanoparticles were toxic to oocytes and inhibited the maturation process (Figure 13A) [35]. In case of AuAg alloy nanoparticles, oocyte maturation was critically impaired at a GMF of 0.2, while for GMF > 0.2 no significant influence was found (Figure 13A). Even though the fact that nanoparticles with higher GMF are less toxic seems rather intuitive, interestingly the bio-response of the AuAg alloy nanoparticle seemed to be non-linearly correlated with their composition. Hence at high GMF the gold seems to passivate the particles while adverse effects strongly increase as a certain threshold in the composition of the particle is reached. These findings could be reproduced by Grade et al. [148] who also found a steep increase of cytotoxicity as well as antimicrobial effects at GMF < 0.5 (Figure 13B). In many studies, toxicity of silver nanoparticles is reported to be linked to Ag^+^ ion release [149–150], which may be a reasonable assumption for AuAg alloy nanoparticles. This hypothesis is backed by data from Besner and Meunier [29] who reported the significant onset of particle dissolution at GMF < 0.4, the same GMF regime where toxic effects started to emerge. Other studies conducted by Alissawi et al. [151] showed that significant ion burst release already appeared at GMF ≈ 0.5, explained by the fact that entropic effects particularly favor a composition of GMF = 0.5. However, these experiments were made with nanocomposites and do not necessarily apply to colloidal nanoparticles. Furthermore, it could be demonstrated that the cytotoxicity of AuAg alloy nanoparticles is likewise affected by the presence of surface ligands [148]. Here, citrate reduced cytotoxic and antimicrobial effects, while they were both more pronounced in the presence of albumin. These findings may be attributed to the reduction of silver ions by surface bound citrate. The latter is frequently applied during the synthesis of silver nanoparticles [152–153], which in this case may reduce ion release and hence toxicity. In addition to ion release, the surface chemistry of the nanoparticle itself may also be directly associated with nanotoxicological effects, e.g., the formation of reactive oxygen species [33]. Here, surface atoms may trigger chemical reactions with biomolecules which are possibly harmful to the organism. These processes are favored by crystal defects, UV light activation and composition and oxidation state of the surface of the nanoparticle. Hence toxicity of nanoparticles may be dominated by its catalytic activity at the nano–bio interface [36]. Even though these findings were predominantly applied for a systematic evaluation of adverse effects of less noble metals and metal oxide nanoparticles [154], their applicability to AuAg alloys may still be possible. The driving forces in this context were identified to be the surface potential and oxidation state of the nanoparticles, both potentially relevant in AuAg nanoparticles, composed of two metals with deviating redox potentials. Additionally, it should be noted that ion release and oxidative stress are not necessarily independent. For example, in the case of ZnO nanoparticles the formation of ROS due to released Zn^2+^ ions was reported to be the dominating mechanism involved in nanotoxicity of these materials [82–83].

Next to the above specified examples on AuAg alloys [35,148] the applicability of ligand-free Au and Ag nanoparticles in reproduction biology was demonstrated. This includes effects on spermatozoa [147] as well as on embryo development [155]. However, utilization of laser-fabricated nanomaterials is not limited to this field but these materials were also applied in a multitude of other toxicological trials. Nanoparticles from laser-based synthesis were frequently used in in vitro assays where adverse effects of Au, Ag, Co, Cu [156], TiO_2_, ZnO [157], Ni, NiTi, NiFe, Ti [11] and MoS_2_ [158] on the viability of mammalian cell lines were studied. Furthermore, some studies addressed antimicrobial effects of Ni [159] and Ag nanoparticles [160–161]. Additionally, some authors reported on the application of laser-fabricated CuO [162], Ag and Au [163] nanoparticles in in vivo trials on rats. Details related to all the above mentioned studies are summarized in Table 2.

**Table 2:** Application of ligand-free nanomaterials obtained from PLAL in toxicological assays.

Nanomaterial	Biological function	Target species	Reference

Au, Ag, AuAg	Functional toxicity/reproduction biology	Oocytes, spermatozoa	[35]
Au, Ag, AuAg	Antimicrobial/in vitro toxicity	*S. aureus*/human fibroblasts	[148]
Au	Functional toxicity/reproduction biology	spermatozoa	[147]
Au, Ag	Embryo development/reproduction biology	Murine embryos	[155]
Au, Ag, Co, Cu	In vitro toxicity	Human cancer cell lines (HeLa, PC3, MCF-7)	[156]
Au, Ag, TiO_2_, ZnO	In vitro toxicity	Human cancer cell lines (HeLa, PC3, MCF-7)	[157]
Ni, NiFe, NiTi, Co, Ti	In vitro toxicity	Human endothelial cells/ Human smooth muscle cells	[11]
MoS_2_	In vitro toxicity	Human CCC-ESF-1, K562 and A549 cells	[158]
Ni	Antimicrobial	*E. coli*	[159]
AgCl	Antimicrobial	*E. coli*	[160]
Ag	Antimicrobial	*S. aureus*	[161]
CuO	In vitro toxicity	Rat model: organ accumulation, systemic toxicity	[162]
Ag, Au	In vitro toxicity	Rat model: inflammation, cellular uptake, systemic toxcicity, genotoxicity	[163]

## Conclusion

Risk assessment in medical implant approval requires the quantification of unintended effects related to wear debris on the nanoscale. Hence, suitable nanoparticle reference materials are needed which have to be totally ligand-free in order to avoid cross contaminations. Such demands are ideally fulfilled by nanoparticles obtained by pulsed laser ablation in liquid (PLAL). Size control of these materials without artificial stabilizers can be achieved via pulsed laser fragmentation in liquids (PLFL), in situ size quenching by electrolytes, delayed conjugation in liquid flow and pulsed laser melting in liquids (PLML). For gold as an exemplary inert noble metal, these methods allow for the tuning of the particle diameters in the range 4–400 nm. However, wear debris predominantly consists of alloy nanoparticles, hence particle composition needs to be considered as well. Here PLAL gives access to reference materials composed of binary and ternary implant alloy nanoparticles with compositions basically representing the bulk materials and homogeneous ultrastructures. However, exceptions may occur in materials with strongly deviating oxygen affinities like NiTi. Furthermore, particle composition may be controlled by laser-based post synthesis alloying or PLAL of representative alloy targets, which could be demonstrated for an AuAg model system. Altogether, PLAL gives access to totally ligand-free alloy nanoparticles with controlled composition and totally homogeneous ultrastructures, which rule out cross effects from elemental segregation on a single-particle level as well as from unwanted surface ligands. However, the fate of these materials in biological systems is influenced by interactions with medium components, predominantly electrostatic effects due to high salinity and electrosteric forces by protein corona formation, factors which need to be considered in all bio-response studies. Exemplary applications of laser-fabricated AuAg alloy nanoparticles in toxicity assays with bacteria as well as mammalian fibroblasts and gametes reveal distinctive correlations between particle composition and toxic effects. Due to the high purity of the material the influence of additional surface ligands could be systematically studied. High purity of these reference materials is of paramount importance in reproduction biology, as fertilization is a highly sensitive process where a single cell counts.
